# Derivation of Embryonic Stem Cells from an Endangered Cattle Breed via Somatic Cell Nuclear Transfer

**DOI:** 10.3390/cells15070627

**Published:** 2026-03-31

**Authors:** Shigang Gu, Xinhua Wei, Yurong Zhang, Jinqian Wang, Lu Tang, Wenxuan Zhao, Jing Wang, Yongye Huang

**Affiliations:** 1 State Key Laboratory of Animal Genetics, Breeding and Reproduction of Ministry of Agriculture and Rural Affairs, Institute of Animal Science, Chinese Academy of Agricultural Sciences, Beijing 100193, China; 18943912896@163.com (S.G.); weixh1204@163.com (X.W.); 15736705130@163.com (Y.Z.); 15114781228@163.com (J.W.); 15730659531@163.com (L.T.); wxzhao9927@163.com (W.Z.); wangjing15@caas.cn (J.W.); 2 Academy of Animal Science, Xizang Agricultural and Animal Husbandry University, Nyingchi 850032, China; 3 National Germplasm Center of Domestic Animal Resources, Institute of Animal Science, Chinese Academy of Agricultural Sciences, Beijing 100193, China; 4 College of Life and Health Sciences, Northeastern University, Shenyang 110169, China

**Keywords:** cattle, fibroblasts, somatic cell nuclear transfer, embryonic stem cells, germplasm resources

## Abstract

**Highlights:**

**What are the main findings?**

- We have successfully generated and maintained stable embryonic stem cell lines (APNT-ESCs) from somatic cell nuclear transfer embryos using fibroblasts of the endangered *ApèiJiaza* cattle.
- The APNT-ESCs exhibited core characteristics of pluripotency, including high expression of key markers, genomic stability, and the capacity to differentiate into cell types representing all three germ layers in vitro.

**What are the implications of the main findings?**

- This work provides a practical and effective biotechnological solution for preserving the genetic resources of endangered species when gametes are inaccessible.
- It establishes a foundational framework for conservation cloning, enabling deeper genetic studies and potential future applications for the revitalization and utilization of endangered breeds.

**Abstract:**

Embryonic stem cells represent a valuable germplasm resource with significant implications for breed conservation, development, and utilization. However, the scarcity of genetic resources in endangered species poses a fundamental constraint on obtaining gametes for embryonic stem cell derivation. Therefore, generating embryonic stem cells from somatic cell nuclear transfer blastocysts offers an optimal alternative for conservation cloning. In this study, we established *ApèiJiaza* somatic cell nuclear transfer ESCs (APNT-ESCs) from cloned embryos, using *ApèiJiaza* cattle ear fibroblasts as nuclear donors. APNT-ESCs could be passaged for over 30 generations in vitro, exhibiting high expression of key pluripotency markers, genomic stability, and the ability to form embryoid bodies and differentiate into cell types of all three germ layers. This research established an effective biotechnological framework for the genetic conservation of other endangered species lacking accessible gametes.

## 1. Introduction

Stem cells, capable of unlimited self-renewal and differentiation into various cell types, including germ cells, represent a powerful tool for genetic conservation. Currently, induced pluripotent stem cells (iPSCs) have been the primary stem cell technology applied to endangered species, as reported in species such as the northern white rhinoceros (*Ceratotherium simum* cottoni), Grevy’s zebra (*Equus grevyi*), Tasmanian devil (*Sarcophilus harrisii*), Sumatran rhinoceros (*Dicerorhinus sumatrensis*), *giant panda*, and *wild boar* [1,2,3,4,5,6]. However, iPSC reprogramming can give rise to copy number variations (CNVs) [7,8], protein-coding mutations [9], and epigenetic defects in DNA methylation and gene expression at imprinting and X-chromosome inactivation regions [10,11,12], all of which may hinder their further application in animals, although these potential limitations of iPSCs have generally been addressed in human studies [13,14,15]. For individuals from whom embryonic stem cells (ESCs) cannot be derived, an alternative approach to obtaining autologous pluripotent stem cells is through nuclear transfer embryonic stem cells (NT-ESCs) generated from somatic cell nuclear transfer (SCNT) blastocysts. NT-ESCs in bovine were first established in 1998 [16], where genetically modified bovine fetal fibroblasts served as donor cells for nuclear transfer, enabling the derivation of embryonic stem (ES)-like cells from cloned blastocysts. Upon reintroduction into preimplantation embryos, these cells differentiated into fetal tissues representing all three germ layers, demonstrating that nuclear transfer can reprogram fibroblasts into an ESC-like state. Crucially, the derivation of NT-ESCs without exogenous genetic manipulation offers a uniquely powerful strategy for generating autologous stem cell lines from elite genotypes, preserving their genetic integrity for future applications. After that, NT-ESCs have been systematically studied in mice [17,18,19], non-human primates [20,21], and humans [22,23,24], with additional reports in porcine [25] and bovine models [16,26]. Subsequent research in mice and humans has shown that NT-ESCs exhibit epigenetic states closer to those of natural ESCs than iPSCs do, suggesting that they may serve as an optimal substitute for ESCs [27,28].

*ApèiJiaza* cattle, a dual-purpose dairy and beef breed from the Tibetan region, have evolved over nearly a millennium to develop a unique gene pool adapted to high-altitude environments [29,30]. Systematic study of this species can not only advance our understanding of the mechanisms underlying plateau adaptation, but also contribute to the breeding of new varieties. However, according to China’s third national survey of genetic resources in 2021, only 39 individuals remained, with just one breeding bull available, placing the species on the brink of functional extinction. Although conservation efforts have been initiated, conventional breeding and species preservation techniques face significant limitations in maintaining the breed’s genetic diversity. There is an urgent need to apply advanced technologies to preserve this valuable genetic resource.

In this study, ear margin fibroblast cells from *ApèiJiaza* cattle were isolated and used as nuclear donors for somatic cell nuclear transfer to generate cloned blastocysts, and APNT-ESCs lines were successfully established. To our knowledge, this is the first application of NT-ESC technology for the conservation of an endangered species. Through our characterization, we confirmed the pluripotent state and in vitro differentiation capacity of APNT-ESCs. In the future, APNT-ESCs may serve as critical material for trait research and population restoration, helping enhance the genetic diversity of *ApèiJiaza* cattle.

## 2. Materials and Methods

### 2.1. Acquisition of Donor Fibroblasts

Ear margin tissue samples were collected from a 1.5-year-old male *ApèiJiaza* cattle, in China’s Tibet. After disinfection and vacuum sealing, the sample was refrigerated and transported to the laboratory. The tissue sample was transferred to a culture dish, disinfected by soaking in alcohol, and then rinsed with PBS. The tissue was then placed in another culture dish and minced. Using a pipette, the tissue fragments were inoculated into a culture flask, culture medium was added, and the flask was placed in an incubator for primary culture. When the cell confluence reached over 95%, subculture was performed by detaching the cells and inoculating them into culture dishes. Once the ear margin fibroblasts were passaged to the P4–P6 generation, they were ready to be used as donor cells.

### 2.2. Oocyte In Vitro Maturation

Ovaries from Holstein cattle were collected from local slaughterhouse. Follicular fluid was aspirated from follicles 2–8 mm in diameter on the ovarian surface using a syringe prefilled with 3–5 mL of collection medium (M199 supplemented with 0.1% heparin and 10% fetal bovine serum, FBS). Cumulus–oocyte complexes (COCs) surrounded by at least three layers of compact cumulus cells were selected under a stereomicroscope (Nikon Corporation, Tokyo, Japan). The selected COCs were washed three times in the collection medium and then transferred to maturation medium (M199 supplemented with 10 µg/L estradiol (E2), 10 µg/L follicle-stimulating hormone (FSH), 10 µg/L luteinizing hormone (LH), 5 ng/mL epidermal growth factor (EGF), 100 µg/L insulin-like growth factor (IGF), and 10% FBS) for 22 h of in vitro maturation.

### 2.3. Generation of ApèiJiaza SCNT Embryos

After maturation, the COCs were placed in a solution containing 0.1 mg/mL hyaluronidase to remove the cumulus cells. Matured oocytes with a clear perivitelline space, intact oolemma, homogeneous cytoplasm, and the first polar body extruded were selected and transferred into T10 manipulation droplets (M199 medium supplemented with 10% FBS and 5 μg/mL cytochalasin B). The oocyte nuclei were enucleated, and a single fibroblast from *ApèiJiaza* cattle was injected into the perivitelline space using an injection pipette. The reconstructed cloned embryos were transferred into CR1-B culture medium and placed in an incubator at 38.5 °C, 5% CO_2_, and 100% humidity for recovery for 0.5–1 h. Subsequently, electrical fusion was performed. After fusion, the embryos were returned to the incubator for another 0.5–1 h of recovery, after which fusion success was assessed. All successfully fused embryos were chemically activated by treatment with 5 μM ionomycin for 5 min, followed by incubation in culture medium containing 1.9 mM 6-dimethylaminopurine for 4 h. After activation, the reconstructed embryos were transferred into CR1-B culture medium (containing 110 mM NaCl, 3 mM KCl, 26.2 mM NaHCO_3_, 1.5 mM glucose, 0.5 mM sodium pyruvate, essential amino acids, non-essential amino acids, and 10% bovine serum albumin) for further culture at 38.5 °C, 5% CO_2_, 5% O_2_ and 90% N_2_.

### 2.4. Derivation of APNT-ES Cells

Derivation of APNT-ESCs was performed using Day-7 blastocysts. The zona pellucida of each blastocyst was gently removed with a 30-gauge insulin needle, and the embryo was transferred onto a feeder-coated 12-well culture plate. Using a needle, the embryo was lightly anchored to the plate surface, with one blastocyst placed per well. Culture was maintained in bEPSC medium [31], which was replaced daily for approximately 15 days. Subsequently, cells were dissociated with TrypL™ Express (Thermo Fisher Scientific, Inc., Waltham, MA, USA) and replated onto fresh feeder-coated 12-well plates. Distinct stem-cell colonies typically emerged within about 5 days post replating. Established APNT-ESCs were routinely cultured in bEPSC medium with daily medium changes and passaged with fresh feeder-coated 12-well plates every 3 days at a 1:6 ratio using TrypL™ Express for dissociation. The composition of bEPSC medium was as previously described. bEPSC medium is mTeSR™1-based media (STEMCELL Technologies, Inc., Vancouver, BC, Canada). bEPSC media (50 mL) was prepared as follows: 48.5 mL of mTeSR™1, 0.5 mL of 100× penicillin–streptomycin (Gibco, Waltham, MA, USA), 0.1 mM 2-mercaptoethanol (Thermo Fisher Scientific, Inc., Waltham, MA, USA), and the following small molecules and cytokines: 1 μM CHIR99021 (Selleck Chemicals Co., Ltd., Houston, TX, USA), 0.3 μM WH-4-023 (Selleck Chemicals Co., Ltd., Houston, TX, USA), 5 μM XAV939 (Merck, Darmstadt, Germany) or 5 μM IWR-1 (Selleck Chemicals Co., Ltd., Houston, TX, USA), 50 μg mL^−1^ Vitamin C (Merck, Darmstadt, Germany), 10 ng mL^−1^ LIF (Merck, Darmstadt, Germany), and 20.0 ng mL^−1^ Activin A (R&D Systems, Inc., Minneapolis, MN, USA).

### 2.5. RT-qPCR

Total RNA from APNT-ES cells was extracted using the RNeasy Plus Mini Kit (Qiagen, 74104, Venlo, The Netherlands). cDNA was synthesized via reverse transcription using the HiScript IV 1st Strand cDNA Synthesis Kit (Vazyme Biotech Co., Ltd., Nanjing, China). The PCR reaction mixture was prepared according to the Taq Pro Universal SYBR qPCR Master Mix (Vazyme Biotech Co., Ltd., Nanjing, China), and RT-qPCR was performed using the Quant Studio™ Real-Time PCR System (Thermo Fisher Scientific, Inc., Waltham, MA, USA). All experiments included three biological replicates, and β-ACTIN was used as the reference gene. The relative expression level of the target gene was determined using the 2^−ΔΔCt^ method. Data are shown as the mean and SD. Primer details are provided in Supplementary Table S1.

### 2.6. Immunofluorescence Staining

Following the removal of the culture medium, the cells were briefly rinsed with DPBS and fixed with 4% paraformaldehyde for 30 min at room temperature. Subsequently, cells were permeabilized using DPBS supplemented with 0.1% Triton X-100 for 30 min. This was followed by blocking with DPBS containing 1% BSA for 1 h at room temperature. The cells were then incubated with primary antibodies overnight at 4 °C, followed by three washes of 10 min each with wash buffer (DPBS containing 0.1% Triton X-100 and 0.1% Tween 20). Secondary antibodies were applied and incubated for 1 h at room temperature protected from light. The washing step was repeated three times in the same manner. Cells were stained with DAPI for 5 min, rinsed with DPBS, and photographed using a fluorescence microscope. Details on the antibodies are provided in Supplementary Table S2.

### 2.7. Western Blot

The culture medium was aspirated prior to protein extraction, and cells were rinsed once with DPBS. Following dissociation with TrypL™ Express, cell pellets were collected and lysed using Native Lysis Buffer (Solarbio Biotech Co., Ltd., Nanjing, China) supplemented with Phenylmethylsulfonyl fluoride (Solarbio Biotech Co., Ltd., Nanjing, China) and Protein Phosphatase Inhibitor (Solarbio Biotech Co., Ltd., Nanjing, China). Whole-cell lysates were separated by SDS–PAGE and transferred onto 0.2 μm PVDF membranes (Bio-Rad Laboratories, Inc., Hercules, CA, USA). Membranes were blocked for 1 h at room temperature with TBST buffer containing 5% non-fat milk. Primary antibody incubation was performed overnight at 4 °C, followed by secondary antibody incubation for 1 h at room temperature. Antibody–protein complexes were visualized using BeyoWB™ BeyoECL (Beyotime Biotechnology Co., Ltd., Shanghai, China). Details on the antibodies are provided in Supplementary Table S2.

### 2.8. Alkaline Phosphatase (AP) Staining

APNT-ESCs were plated at low density onto feeder cell-coated 12-well plates and cultured for 5 days, followed by staining with BM Purple AP (F. Hoffmann-La Roche & Co. AG, Basel, Switzerland) according to the manufacturer’s recommended protocol.

### 2.9. Karyotype Analysis

Before karyotype analysis, APNT-ESCs were treated with bEPSC medium supplemented with 0.1 μg/mL colchicine for 2 h. Subsequently, cells were dissociated using TrypL™ Express at 37 °C for 5 min, and cell pellets were collected by centrifugation. Hypotonic treatment was performed with 0.075 M KCl at 37 °C for 30 min, followed by fixation with a 3:1 mixture of methanol and acetic acid for 20 min, repeated three times. Cells were resuspended, dropped onto pre-chilled slides, and allowed to air-dry at room temperature before being stained with Giemsa Stain Solution (Solarbio Biotech Co., Ltd., Nanjing, China). For each slide, more than 30 metaphase spreads were evaluated.

### 2.10. EB Formation

APNT-ESCs were dissociated into single cells and subsequently cultured for 7 days in ultralow-attachment plates using Knockout DMEM (Thermo Fisher Scientific, Inc., Waltham, MA, USA) supplemented with 15% FBS (Thermo Fisher Scientific, Inc., Waltham, MA, USA), 1% penicillin–streptomycin (Thermo Fisher Scientific, Inc., Waltham, MA, USA), and 1% GlutaMAX (Thermo Fisher Scientific, Inc., Waltham, MA, USA). The resulting embryoid bodies (EBs) were then transferred into 12-well plates and 35 mm confocal dishes (Solarbio Biotech Co., Ltd., Nanjing, China) for RT-qPCR analysis and immunofluorescence staining.

### 2.11. CUT&Tag and Data Processing

CUT&Tag was performed using a Hyperactive Universal CUT&Tag Assay Kit (Vazyme). Paired-end sequencing was conducted on an Illumina NovaSeq Xplus system by Beijing Novogene Bioinformatics Technology Co., Ltd., Beijing, China. Paired-end reads were aligned against the bovine reference genome ARS-UCD2.0 using Bowtie2 (v.2.5.4) with the following parameters: −very-sensitive −no-discordant −no-mixed−no-unal-X 2000. For peak calling, MACS2 (v.2.2.9.1) was used with the following parameters: −nomodel−shift 100−extsize 200−keep-dup all.

## 3. Results

### 3.1. Derivation of NT-ESCs from Cloned Blastocysts of ApèiJiaza Cattle

**Figure 1:**
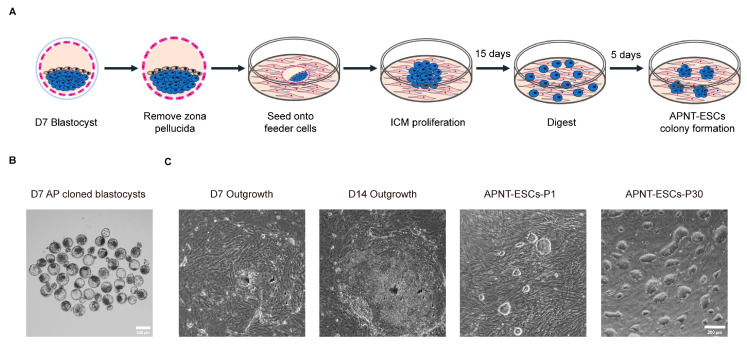
Generation of APNT-ESCs from SCNT blastocysts. (**A**) Strategies for establishment of APNT-ESCs using *ApèiJiaza* cattle cloned embryos. Blue cells represent the inner cell mass and derived stem cells, while pink cells represent the trophoblast cells. (**B**) Morphology of preimplantation development of *ApèiJiaza* cattle cloned embryos at day 7. Scale bar, 200 μm. (**C**) Morphological images of outgrowths and APNT-ESCs. Scale bar, 200 μm.

To establish APNT-ESCs, ear margin tissues were collected from *ApèiJiaza* cattle to derive fibroblast cell lines. Using these fibroblast cells as nuclear donors for somatic cell nuclear transfer, at 24 h following injection, 73 (91.25%) of the reconstructed embryos were observed to have undergone cleavage. We obtained 39 blastocysts (48.75%) from 80 reconstructed embryos in one experiment (Figure 1B), of which 26 morphologically sound blastocysts were selected for APNT-ESC lines derivation (Figure 1A). Approximately 15 days later, 15 APNT-ESC lines were successfully established (Figure 1C). These cells grew as compact, dome-shaped colonies with smooth edges on feeder-coated culture dishes (Figure 1C). Although most lines could not be maintained through long-term passaging, three lines among them remained stable beyond 30 passages (Figure 1C). The derivation efficiency of APNT-ESCs was 11.5%.

### 3.2. Verification of the Pluripotency and In Vitro Differentiation Potential of APNT-ESCs

**Figure 2:**
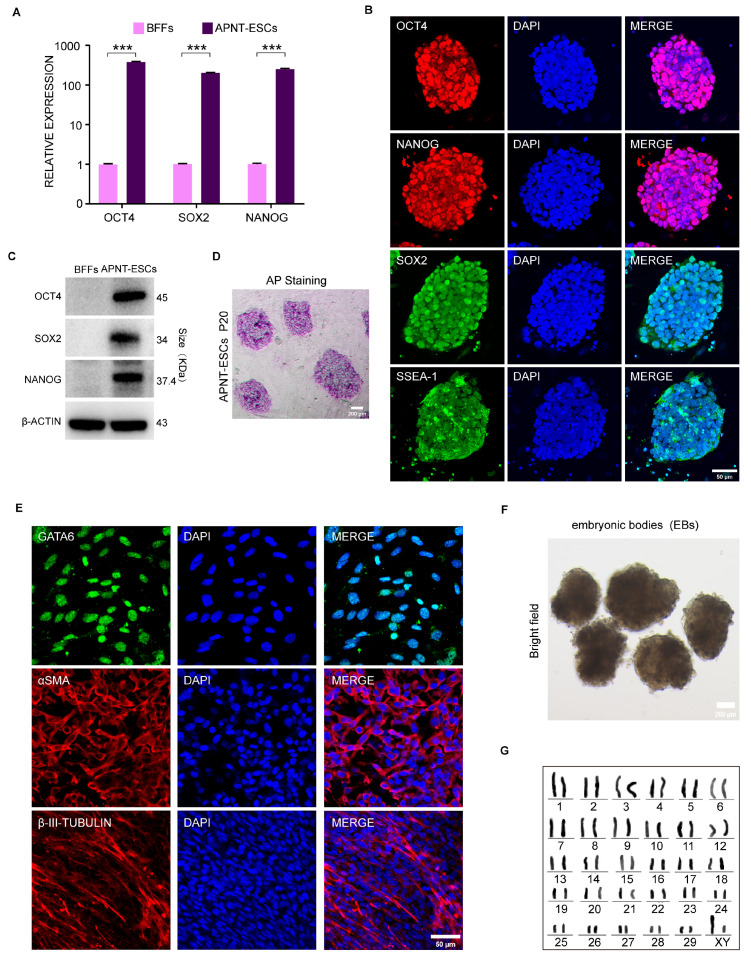
Pluripotency of APNT-ESCs was validated by positive expression of pluripotency markers and robust EB formation capacity. (**A**) The mRNA expression levels of representative pluripotent marker genes in APNT-ESCs. Data represent the mean ± SD; *n* = 3 independent experiments. “*” Indicates the degree of difference. (**B**) Immunostaining of pluripotency markers OCT4, NANOG, and SOX2 in APNT-ESCs. DAPI was used to stain nuclei. Scale bar, 50 μm. (**C**) Western blot analysis of core pluripotency markers (OCT4, SOX2, and NANOG) in APNT-ESCs. *n* = 3 independent experiments with similar results. (**D**) Morphology and alkaline phosphatase (AP) staining of APNT-ESCs. Scale bar, 200 μm. (**E**) Immunostaining for ectodermal neuro-specific marker protein β-III-TUBULIN, mesodermal muscle-specific marker protein α-SMA and endodermal-specific marker protein GATA6. DAPI was used for staining nuclei. Scale bar, 50 μm. (**F**) Morphology of embryonic bodies (EBs) derived from APNT-ESCs. *n* = 3 independent experiments with similar results. Scale bar, 200 μm. (**G**) Karyotype analyses of APNT-ESCs. For each cell line, 45 cells at metaphase were examined.

The expressions of pluripotency genes of APNT-ESCs were determined. RT-qPCR analysis showed that the expression levels of the core pluripotency genes *OCT4*, *SOX2*, and *NANOG* were significantly higher in APNT-ESCs than in control APJZ fibroblasts (Figure 2A). Consistent results were obtained at the protein level by immunofluorescence staining and Western blot analysis (Figure 2B,C). Moreover, APNT-ESC colonies stained positively for alkaline phosphatase (AP) (Figure 2D). In addition, an in vitro differentiation assay revealed positive staining of GATA6 (endoderm), α-SMA (mesoderm), and βIII tubulin (ectoderm) (Figure 2E). It also evidenced that APNT-ESCs possess the capability to form embryonic bodies and differentiate into all three germ layers (Figure 2F). Karyotype analysis confirmed that APNT-ESCs maintain normal cytogenetic profiles throughout extended culture (Figure 2G).

### 3.3. Chromatin State on the Pluripotency Genes of APNT-ESCs

**Figure 3:**
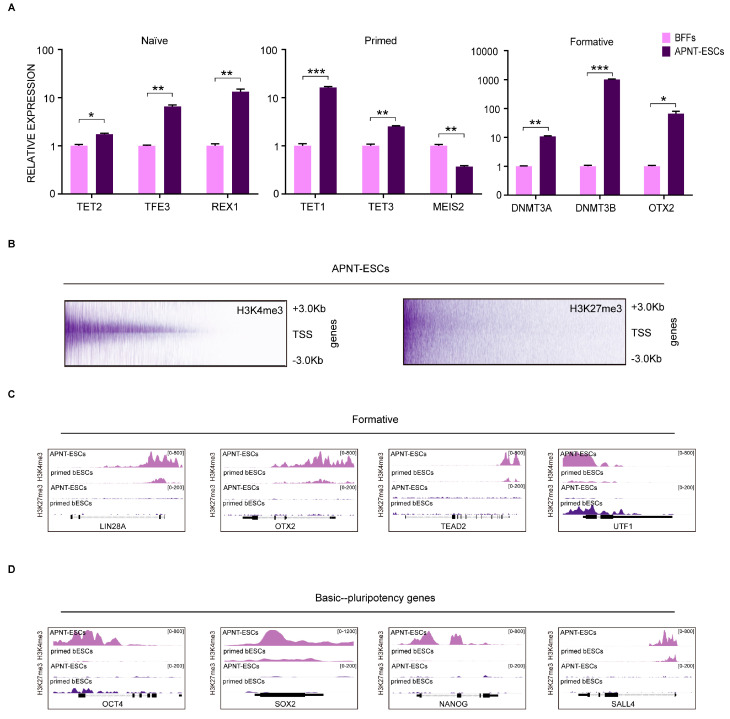
Analysis of chromatin state of APNT-ESCs. (**A**) The mRNA expression levels of representative naïve, primed and formative marker genes in APNT-ESCs. Data represent the mean ± SD; *n* = 3 independent experiments. “*” Indicates the degree of difference. (**B**,**C**) H3K4me3 and H3K27me3 tracks of representative formative pluripotency genes in APNT-ESCs and primed bESCs. (**D**) H3K4me3 and H3K27me3 tracks of basic-pluripotency genes in APNT-ESCs and primed bESCs.

To uncover the mechanisms in shaping the pluripotency state of APNT-ESCs, we examined the expression of stage-specific marker genes (naïve: *TEAD4*, *TET2*, and *TFE3*; formative: *OTX2*, *DNMT3A*, and *DNMT3B*; and primed: *TET1*, *TET3*, and *MEIS2*) [31,32,33]. The results indicated a pronounced up-regulation of formative-associated genes in APNT-ESCs (Figure 3A). To further characterize the epigenetic landscape, we performed Cleavage Under Targets and Tagmentation (CUT&Tag) assays (Figure 3B). Compared with primed bovine ESCs (GEO: PRJNA432599) [32], APNT-ESCs exhibited higher H3K4me3 and lower H3K27me3 abundance at the promoters of formative-related genes (Figure 3C). Similarly, the chromatin marks of core pluripotency genes in APNT-ESCs embodied increased H3K4me3 and decreased H3K27me3, especially for *OCT4* and *NANOG* (Figure 3D). Based on these findings, we preliminarily classify APNT-ESCs as residing in a formative pluripotency state.

## 4. Discussion

NT-ESC technology holds a unique technical position in the context of endangered animal research. In 1998, following the first successful establishment of a nuclear transfer embryonic stem cell line in cattle [16], researchers began to delve deeper into the study and discussion of NT-ESCs. In the early 2000s, research in mice progressed from establishing pluripotent ES-like cell lines from blastocysts reprogrammed by somatic cell nuclear transfer to large-scale production and systematic pluripotency validation of NT-ESCs and, finally, to establishing a paradigm for NT-ESCs-based therapeutic models in mice—involving NT-ESC line derivation, gene correction, and subsequent transplantation therapy [17,18,34]. Later, researchers discovered that ESCs and NT-ESCs are highly similar in terms of transcriptome and pluripotency [35], implying that pluripotent stem cells free from exogenous factor integration could be established without the use of gametes. Following the ascendance of iPSC technology, research into nuclear transfer embryonic stem cells (NT-ESCs) comparatively decreased. iPSCs have predominantly been employed for germplasm conservation of endangered species through stem cell-based approaches, primarily due to the inability to collect gametes or obtain embryos for embryonic stem cell (ESC) derivation [1,4,36]. However, iPSC generation involves the introduction of exogenous genes and often results in incomplete reprogramming, leading to aberrant epigenetic states [37,38]. These limitations have been systematically demonstrated in mice and humans and may significantly compromise the functional utility of such cells for downstream applications.

In previous reports on bovine NT-ESCs, Wu et al., by investigating the role of CDX2 in inducing the trophoblast lineage in the bovine inner cell mass, found that CDX2 knockout enables nuclear transfer-derived bovine embryonic stem cells to stabilize more easily and better maintain pluripotency [39]. Other researchers have examined the efficiency of establishing embryonic stem cell lines from bovine blastocysts at different stages, providing insights into the sources of cellular phenotypic changes under bESCs culture conditions and offering new perspectives on the derivation, maintenance, and characterization of ESCs [40]. In addition, some research groups have developed different culture systems for bovine embryonic stem cells, such as CTFR and bEPSCM, and successfully derived nuclear transfer embryonic stem cell lines from somatic cell nuclear transfer blastocysts using these systems [31,32]. These studies have advanced the development of bovine NT-ESCs; however, research focusing on endangered species conservation and the establishment of high-quality nuclear transfer embryonic stem cell lines remains limited to date.

In this study, we established NT-ESCs from the endangered *ApèiJiaza* cattle through SCNT-mediated embryonic reprogramming. The derivation process of these cell lines largely mirrors that of conventional ESCs and does not rely on exogenous gene expression. Our findings align with earlier reports in other species, further confirming that SCNT can reprogram terminally differentiated somatic cells into a highly plastic pluripotent state. However, due to limitations in sample collection, we were only able to derive APNT-ES cell lines from a single individual. Although this cannot represent the broader genetic profile of the *ApèiJiaza* cattle population, it still constitutes a meaningful contribution given the current endangered status of this breed. In fact, not all APNT-ES cell lines could be maintained through long-term passaging; most of the cell lines lost their clonal morphology during passaging. This issue may be attributed to differences among donor cells [41,42]. Although all donor cells were derived from the same individual, some cells inevitably underwent senescence or exhibited epigenetic abnormalities during in vitro culture [43]. This influence could not be completely eliminated during donor cell selection, potentially contributing to variability among SCNT embryos and ultimately leading to the instability observed in the derived stem cell lines. At the level of pluripotency gene expression, APNT-ESCs exhibited high expression of the core pluripotency genes *OCT4*, *SOX2* and *NANOG*. In addition, they also showed high expression of the surface marker SSEA1. Saito et al. reported positive expression of SSEA1 in bovine pluripotent stem cells [44], whereas Wang et al. claimed its expression to be negative [45]. This discrepancy may be attributed to differences in embryonic stages and culture systems. Unlike in mice, where SSEA1 is expressed exclusively in the ICM of blastocysts, a study demonstrated that SSEA1 is expressed in both the ICM and TE of bovine blastocysts [46]. Possibly, its expression level can only serve as a reference and should not be considered a definitive marker for bovine pluripotent stem cells. Overall, our study successfully generated APNT-ESC lines from the functionally endangered *ApèiJiaza* cattle and systematically characterized their basic properties, pluripotency status, and differentiation potential. This work not only provides critical cellular resources for conserving the unique high-altitude adaptive germplasm of *ApèiJiaza* cattle but also explores a feasible stem cell-based pathway for protecting other endangered species from gametes or embryos that are difficult to obtain.

## 5. Conclusions

This study successfully demonstrates the feasibility of generating stable embryonic stem cells from somatic cell nuclear transfer embryos of the endangered *ApèiJiaza* cattle, thereby establishing a critical living germplasm resource for this breed. The research thus provides a validated and effective biotechnological framework for the genetic conservation of other endangered species facing similar constraints due to the scarcity of gametes, opening new avenues for conservation cloning, fundamental research, and potential future applications.

## Data Availability

The datasets used and/or analyzed during the current study are available from the corresponding author upon reasonable request.

## Supplementary Materials

The following supporting information can be downloaded at: https://www.mdpi.com/article/10.3390/cells15070627/s1. **Table S1**: The RT-qPCR primer sequences used in this study. **Table S2**: The antibody information used in this study.

## References

[1] Ben-Nun I.F., Montague S.C., Houck M.L., Tran H.T., Garitaonandia I., Leonardo T.R., Wang Y.C., Charter S.J., Laurent L.C., Ryder O.A. (2011). Induced pluripotent stem cells from highly endangered species. Nat. Methods.

[2] Weeratunga P., Shahsavari A., Ovchinnikov D.A., Wolvetang E.J., Whitworth D.J. (2018). Induced Pluripotent Stem Cells from a Marsupial, the Tasmanian Devil (*Sarcophilus harrisii*): Insight into the Evolution of Mammalian Pluripotency. Stem Cells Dev..

[3] Endo Y., Kamei K.I., Hasegawa K., Okita K., Ito H., Terada S., Inoue-Murayama M. (2022). Generation and Gene Expression Profiles of Grevy’s Zebra Induced Pluripotent Stem Cells. Stem Cells Dev..

[4] Zywitza V., Frahm S., Krüger N., Weise A., Göritz F., Hermes R., Holtze S., Colleoni S., Galli C., Drukker M. (2022). Induced pluripotent stem cells and cerebral organoids from the critically endangered *Sumatran rhinoceros*. iScience.

[5] Liu Y., Zhang S., Zou G., An J., Li Y., Lin D., Wang D., Li Y., Chen J., Feng T. (2024). Generation and characterization of giant panda induced pluripotent stem cells. Sci. Adv..

[6] Gao C., Zhou X., Gu S., Li J., Wang J., Zhang Y., Zhao W., Wei X., Lu L., Zhao Q. (2025). Establishment of Interspecies Somatic Cell Nuclear Transfer and Transgene-Free Inducible Pluripotent Stem Cells for Versatile Conservation of the Germplasm Resource of Wild Boar. Anim. Res. One Health.

[7] Hussein S.M., Batada N.N., Vuoristo S., Ching R.W., Autio R., Närvä E., Ng S., Sourour M., Hämäläinen R., Olsson C. (2011). Copy number variation and selection during reprogramming to pluripotency. Nature.

[8] Laurent L.C., Ulitsky I., Slavin I., Tran H., Schork A., Morey R., Lynch C., Harness J.V., Lee S., Barrero M.J. (2011). Dynamic changes in the copy number of pluripotency and cell proliferation genes in human ESCs and iPSCs during reprogramming and time in culture. Cell Stem Cell.

[9] Ruiz S., Gore A., Li Z., Panopoulos A.D., Montserrat N., Fung H.L., Giorgetti A., Bilic J., Batchelder E.M., Zaehres H. (2013). Analysis of protein-coding mutations in hiPSCs and their possible role during somatic cell reprogramming. Nat. Commun..

[10] Nazor K.L., Altun G., Lynch C., Tran H., Harness J.V., Slavin I., Garitaonandia I., Müller F.J., Wang Y.C., Boscolo F.S. (2012). Recurrent variations in DNA methylation in human pluripotent stem cells and their differentiated derivatives. Cell Stem Cell.

[11] Ohi Y., Qin H., Hong C., Blouin L., Polo J.M., Guo T., Qi Z., Downey S.L., Manos P.D., Rossi D.J. (2011). Incomplete DNA methylation underlies a transcriptional memory of somatic cells in human iPS cells. Nat. Cell Biol..

[12] Ruiz S., Diep D., Gore A., Panopoulos A.D., Montserrat N., Plongthongkum N., Kumar S., Fung H.L., Giorgetti A., Bilic J. (2012). Identification of a specific reprogramming-associated epigenetic signature in human induced pluripotent stem cells. Proc. Natl. Acad. Sci. USA.

[13] Cantone I., Bagci H., Dormann D., Dharmalingam G., Nesterova T., Brockdorff N., Rougeulle C., Vallot C., Heard E., Chaligne R. (2016). Ordered chromatin changes and human X chromosome reactivation by cell fusion-mediated pluripotent reprogramming. Nat. Commun..

[14] Ruiz S., Lopez-Contreras A.J., Gabut M., Marion R.M., Gutierrez-Martinez P., Bua S., Ramirez O., Olalde I., Rodrigo-Perez S., Li H. (2015). Limiting replication stress during somatic cell reprogramming reduces genomic instability in induced pluripotent stem cells. Nat. Commun..

[15] Kang X., Yu Q., Huang Y., Song B., Chen Y., Gao X., He W., Sun X., Fan Y. (2015). Effects of Integrating and Non-Integrating Reprogramming Methods on Copy Number Variation and Genomic Stability of Human Induced Pluripotent Stem Cells. PLoS ONE.

[16] Cibelli J.B., Stice S.L., Golueke P.J., Kane J.J., Jerry J., Blackwell C., Ponce de León F.A., Robl J.M. (1998). Transgenic bovine chimeric offspring produced from somatic cell-derived stem-like cells. Nat. Biotechnol..

[17] Munsie M.J., Michalska A.E., O’Brien C.M., Trounson A.O., Pera M.F., Mountford P.S. (2000). Isolation of pluripotent embryonic stem cells from reprogrammed adult mouse somatic cell nuclei. Curr. Biol..

[18] Wakayama T., Tabar V., Rodriguez I., Perry A.C., Studer L., Mombaerts P. (2001). Differentiation of embryonic stem cell lines generated from adult somatic cells by nuclear transfer. Science.

[19] Markoulaki S., Meissner A., Jaenisch R. (2008). Somatic cell nuclear transfer and derivation of embryonic stem cells in the mouse. Methods.

[20] Byrne J.A., Pedersen D.A., Clepper L.L., Nelson M., Sanger W.G., Gokhale S., Wolf D.P., Mitalipov S.M. (2007). Producing primate embryonic stem cells by somatic cell nuclear transfer. Nature.

[21] Sparman M., Dighe V., Sritanaudomchai H., Ma H., Ramsey C., Pedersen D., Clepper L., Nighot P., Wolf D., Hennebold J. (2009). Epigenetic reprogramming by somatic cell nuclear transfer in primates. Stem Cells.

[22] Tachibana M., Amato P., Sparman M., Gutierrez N.M., Tippner-Hedges R., Ma H., Kang E., Fulati A., Lee H.S., Sritanaudomchai H. (2013). Human embryonic stem cells derived by somatic cell nuclear transfer. Cell.

[23] Yamada M., Johannesson B., Sagi I., Burnett L.C., Kort D.H., Prosser R.W., Paull D., Nestor M.W., Freeby M., Greenberg E. (2014). Human oocytes reprogram adult somatic nuclei of a type 1 diabetic to diploid pluripotent stem cells. Nature.

[24] Noggle S., Fung H.L., Gore A., Martinez H., Satriani K.C., Prosser R., Oum K., Paull D., Druckenmiller S., Freeby M. (2011). Human oocytes reprogram somatic cells to a pluripotent state. Nature.

[25] Kim S., Kim J.H., Lee E., Jeong Y.W., Hossein M.S., Park S.M., Park S.W., Lee J.Y., Jeong Y.I., Kim H.S. (2010). Establishment and characterization of embryonic stem-like cells from porcine somatic cell nuclear transfer blastocysts. Zygote.

[26] Soto D.A., Navarro M., Ross P.J. (2023). Derivation of Bovine Primed Embryonic Stem Cells from Somatic Cell Nuclear Transfer Embryos. Methods Mol. Biol..

[27] Ma H., Morey R., O’Neil R.C., He Y., Daughtry B., Schultz M.D., Hariharan M., Nery J.R., Castanon R., Sabatini K. (2014). Abnormalities in human pluripotent cells due to reprogramming mechanisms. Nature.

[28] Kim K., Doi A., Wen B., Ng K., Zhao R., Cahan P., Kim J., Aryee M.J., Ji H., Ehrlich L.I. (2010). Epigenetic memory in induced pluripotent stem cells. Nature.

[29] Tang J., Chen X., Song T., Zhao L., Ma J. (2016). The Research on Conservation and Utility of Tibet Cattle Germplasm Resources. China Cattle Sci..

[30] Longgang M., Nan L., Qunzong N.M., Xin W., Jian L., Huaming M., Gong C., Ouzhu D.Z., Ciren L.B., Nan Z. (2024). Study on the Genetic Composition of Four Local Tibetan Cattle Breeds Based on SNP Chip Analysis. Acta Vet. Zootech. Sin..

[31] Zhao L., Gao X., Zheng Y., Wang Z., Zhao G., Ren J., Zhang J., Wu J., Wu B., Chen Y. (2021). Establishment of bovine expanded potential stem cells. Proc. Natl. Acad. Sci. USA.

[32] Bogliotti Y.S., Wu J., Vilarino M., Okamura D., Soto D.A., Zhong C., Sakurai M., Sampaio R.V., Suzuki K., Izpisua Belmonte J.C. (2018). Efficient derivation of stable primed pluripotent embryonic stem cells from bovine blastocysts. Proc. Natl. Acad. Sci. USA.

[33] Yang L., Di A., Song L., Liu X., Wu D., Wang S., Hao Z., Bu L., Bai C., Su G. (2025). Generation of modified cows and sheep from spermatid-like haploid embryonic stem cells. Nat. Biotechnol..

[34] Rideout W.M., Hochedlinger K., Kyba M., Daley G.Q., Jaenisch R. (2002). Correction of a genetic defect by nuclear transplantation and combined cell and gene therapy. Cell.

[35] Brambrink T., Hochedlinger K., Bell G., Jaenisch R. (2006). ES cells derived from cloned and fertilized blastocysts are transcriptionally and functionally indistinguishable. Proc. Natl. Acad. Sci. USA.

[36] Verma R., Holland M.K., Temple-Smith P., Verma P.J. (2012). Inducing pluripotency in somatic cells from the snow leopard (*Panthera uncia*), an endangered felid. Theriogenology.

[37] Edwards M.M., Wang N., Massey D.J., Bhatele S., Egli D., Koren A. (2024). Incomplete reprogramming of DNA replication timing in induced pluripotent stem cells. Cell Rep..

[38] Parikh C., Glenn R.A., Shi Y., Chatterjee K., Kasliwal K., Swanzey E.E., Singer S., Do S.C., Zhan Y., Furuta Y. (2025). Genetic variation modulates susceptibility to aberrant DNA hypomethylation and imprint deregulation in naive pluripotent stem cells. Stem Cell Rep..

[39] Wu X., Song M., Yang X., Liu X., Liu K., Jiao C., Wang J., Bai C., Su G., Liu X. (2016). Establishment of bovine embryonic stem cells after knockdown of CDX2. Sci. Rep..

[40] Guiltinan C., Botigelli R.C., Candelaria J.I., Smith J.M., Arcanjo R.B., Denicol A.C. (2025). Primed bovine embryonic stem cell lines can be derived at diverse stages of blastocyst development with similar efficiency and molecular characteristics. Biol. Open.

[41] Jiao D., Cheng W., Zhang X., Zhang Y., Guo J., Li Z., Shi D., Xiong Z., Qing Y., Jamal M.A. (2021). Improving porcine SCNT efficiency by selecting donor cells size. Cell Cycle.

[42] Whitworth K.M., Prather R.S. (2010). Somatic cell nuclear transfer efficiency: How can it be improved through nuclear remodeling and reprogramming?. Mol. Reprod. Dev..

[43] Armstrong L. (2012). Epigenetic control of embryonic stem cell differentiation. Stem Cell Rev. Rep..

[44] Saito S., Sawai K., Ugai H., Moriyasu S., Minamihashi A., Yamamoto Y., Hirayama H., Kageyama S., Pan J., Murata T. (2003). Generation of cloned calves and transgenic chimeric embryos from bovine embryonic stem-like cells. Biochem. Biophys. Res. Commun..

[45] Wang L., Duan E., Sung L.Y., Jeong B.S., Yang X., Tian X.C. (2005). Generation and characterization of pluripotent stem cells from cloned bovine embryos. Biol. Reprod..

[46] Pant D., Keefer C.L. (2009). Expression of pluripotency-related genes during bovine inner cell mass explant culture. Cloning Stem Cells.

